# Neuroblastoma Breakpoint Family 3mer Higher Order Repeats/Olduvai Triplet Pattern in the Complete Genome of Human and Nonhuman Primates and Relation to Cognitive Capacity

**DOI:** 10.3390/genes15121598

**Published:** 2024-12-13

**Authors:** Matko Glunčić, Ines Vlahović, Marija Rosandić, Vladimir Paar

**Affiliations:** 1 Faculty of Science, University of Zagreb, 10000 Zagreb, Croatia; matko@phy.hr (M.G.); vpaar@hazu.hr (V.P.); 2 Department of Interdisciplinary Sciences, Algebra University College, 10000 Zagreb, Croatia; 3 Department of Internal Medicine, University Hospital Centre Zagreb, 10000 Zagreb, Croatia; rosandic@hazu.hr; 4 Croatian Academy of Sciences and Arts, 10000 Zagreb, Croatia

**Keywords:** NBPF repeats, Olduvai triplet, higher-order repeats, global repeat map (GRM), evolution

## Abstract

Background/Objectives: The ~1.6 kb NBPF repeat units in neuroblastoma breakpoint family (NBPF) genes are specific to humans and are associated with cognitive capacity in higher primates. While the number of NBPF monomers/Olduvai sequences in humans is approximately 2–3 times greater than in great apes, the difference in copy number values of canonical NBPF 3mer Higher-order repeats (HORs)/Olduvai triplets between humans and great apes is substantially larger. This study aims to analyze the organization and evolutionary significance of NBPF 3mer HORs/Olduvai triplets in fully sequenced primate genomes. Methods: We applied the global repeat map (GRM) algorithm to identify canonical and variant NBPF 3mer HORs/Olduvai triplets in the complete genomes of humans, chimpanzees, gorillas, and orangutans. The resulting monomer arrays were analyzed using the GRMhor algorithm to generate detailed schematic representations of NBPF HOR organization. Results: The analysis reveals a distinct difference in NBPF-related patterns among these primates, particularly in the number of tandemly organized canonical 3mer HORs/Olduvai triplets: 61 tandemly organized canonical NBPF 3mer HORs/Olduvai triplets in humans, compared to 0 in chimpanzees and orangutans, and 9 in gorillas. When considering only tandemly organized 3mer HORs/Olduvai triplets with more than three copies, the numbers adjust to 36 in humans and 0 in great apes. Furthermore, the divergence between individual NBPF monomers in humans and great apes is twice as high as that observed within great apes. Conclusions: These findings support the hypothesis that the tandem organization of NBPF 3mer HORs/Olduvai triplets plays a crucial role in enhancing cognitive capacity in humans compared to great apes, potentially providing a significant evolutionary advantage. This effect complements the impact of the increased number of individual NBPF monomers/Olduvai sequences, together contributing to a synergistic amplification effect.

## 1. Introduction

Neuroblastoma is a solid malignancy primarily affecting children [1,2]. The neuroblastoma breakpoint family (NBPF) was identified through the disruption of one of its members in a neuroblastoma patient [3]. The NBPF genes, located in human chromosome 1, contain a repetitive DNA structure composed of ~1.6 kb tandem repeat units [4,5,6]. These repeats encode proteins known as Olduvai domains or NBPF proteins (previously referred to as DUF1220 proteins), which are highly expressed in neocortex neurons in regions critical for advanced cognitive functions. The coding regions of these domains have undergone the largest human-specific increase in copy number within the genome [6,7,8]. Two terminologies have been used to describe the repeat pattern in NBPF genes: the Sikela–van Roy terminology [9] and the Willard terminology [10,11,12] (Table 1). In the Sikela–van Roy framework, the ~1.6 kb tandem repeat units are referred to as Olduvai sequences, whereas, in the Willard framework, they are referred to as NBPF monomers. Higher-order repeats (HORs) comprising three types of ~1.6 kb tandem repeat units are described as Olduvai triplets in Sikela–van Roy terminology or as NBPF 3mer HOR HORs in Willard terminology. The three types of ~1.6 kb repeat units are designated as HLS1, HLS2, and HLS3 (Sikela–van Roy) or as m1, m2, and m3 (Willard).

The Sikela–van Roy terminology was developed specifically for describing tandem repeats in NBPF genes, while the Willard terminology was initially introduced for α satellite repeat units in the centromeric regions of primates. In this framework, HORs were labeled as *n*mer HORs, with the NBPF 3mer HORs later identified [13] and subsequently applied to the Neanderthal genome and the first human T2T complete genome assemblies [14].

The Sikela–van Roy terminology was specifically adapted to identify the ~1.6 kb primary repeat units in NBPF genes, which are mostly organized into triplets [7]. Similarly, the Willard terminology was introduced and extensively used for studying the centromeric ~171 bp α satellite repeat units, which are then combined into HORs of chromosome-specific order *n* [11]. Thus, the Sikela–van Roy terminology corresponds to a special case of Willard terminology for *n* = 3. In the global repeat map (GRM) algorithm, however, the order of repeat identification is reversed: first, HORs are identified, and then the primary repeat units are deduced from the HOR structure [13,15]. The advantage of the GRM approach is that the divergence between HORs is much smaller than between the primary repeat units, allowing for higher precision in HOR identification. However, this method only identifies monomer repeat sequences present in HORs, whereas repeat units that are not organized into HORs—such as monomers CON1-3 detected using the Sikela–Van Roy approach—are not identified.

In human NBPF genes, it has been noted that the first Olduvai triplet of each gene is distinct from the subsequent triplets within that gene [7]. Furthermore, the expanded triplets were shown to have arisen from a recombination event involving the small exon of the CON3 subtype [16]. These observations were not identified in the present computation, as the NBPF monomers examined were derived exclusively from strictly periodic HORs. Consequently, exons of the CON1 type were excluded from this analysis, as outlined in Table 1.

The copy number of Olduvai sequences/NBPF monomers has been systematically correlated with various aspects of brain function and pathology, including brain size, cortical neuron number, IQ scores, cognitive aptitude, autism, schizophrenia, microcephaly, macrocephaly, and neuroblastoma [2,3,4,6,8,16,17,18,19,20,21,22,23,24,25,26,27,28,29,30,31].

Olduvai sequences/NBPF monomers are highly expressed in neocortex neurons, particularly in regions believed to be critical for advanced cognitive functions [6,8,20,31,32]. Furthermore, three of the four NBPF genes with the largest human-specific Olduvai sequences/NBPF monomer expansion are located adjacent to three human-specific *NOTCH2NL* genes, playing a role in neuronal output and the expansion of the human cortex [33].

Previous studies report that the copy number of NBPF monomers/Olduvai sequences is highest in the human genome (~300 copies), followed by great apes, with a gradual decrease as phylogenetic distance from humans increases, eventually becoming largely absent in non-mammalian species [6,7,16,21,22,23,34]. However, these reported values depend significantly on the computational methods used, which are closely examined in this study; thus, the copy number values should be interpreted with caution.

In this study, we compare the human-specific increase in the copy number of tandemly organized NBPF 3mer HORs/Olduvai triplets and individually organized NBPF monomers/Olduvai sequences. As a significant advancement over our previous studies [13,14,35], this research represents the first comprehensive analysis of complete genomic sequences from humans and the observed higher primates—chimpanzees, gorillas, and orangutans. Consequently, the final results are free from gaps or missing monomers, ensuring that the number and structure of the identified HORs are determined solely by the divergence threshold, which is also thoroughly examined and discussed.

Based on these observations, we hypothesize that the tandemly organized ~4.8 kb NBPF 3mer HOR/Olduvai triplet copy number may reflect an additional evolutionary signature, complementing the individual ~1.6 kb NBPF monomer/Olduvai sequence number effect to produce a coherent amplification effect.

It is noteworthy that tandemly arranged Olduvai triplets have been reported to undergo post-translational cleavage by furin, with each triplet being cleaved once [31]. This furin-mediated cleavage, combined with the observed strong correlation between Olduvai copy number and brain size, suggests that the primary evolutionary target of selection in humans was the rapid amplification of autonomously functioning Olduvai triplet proteins. These proteins are proposed to be the key active agents underlying Olduvai’s role in the expansion of the human brain [31]. This finding underscores the biological and evolutionary significance of the NBPF triplets, further emphasizing their critical contribution to human cognitive development.

We also analyze the sensitivity of the NBPF monomer/Olduvai sequence count to the divergence threshold used for defining NBPF monomers/Olduvai sequences in recently sequenced complete human genomes, enabled by advancements in sequencing technologies [36,37,38]. Furthermore, we compare the NBPF 3mer HOR/Olduvai triplet copy numbers between humans and great apes, as well as among great ape species. Finally, we examine the differences in NBPF monomer/Olduvai sequence counts between humans and great apes and among different great ape species.

## 2. Materials and Methods

Reference genome sequences for chromosome 1 are freely available on the National Center for Biotechnology Information’s official website:

T2T_CHM13v2.0 https://www.ncbi.nlm.nih.gov/datasets/genome/GCF_009914755.1/, NHGRI_mPanTro3-v2.0_pri https://www.ncbi.nlm.nih.gov/datasets/genome/GCF_028858775.2/, NHGRI_mGorGor1-v2.0_pri https://www.ncbi.nlm.nih.gov/datasets/genome/GCF_029281585.2/, and NHGRI_mPonAbe1-v2.0_pri https://www.ncbi.nlm.nih.gov/datasets/genome/GCF_028885655.2/ (all URLs accessed on 11 December 2024).

In the first step, using the global repeat map (GRM) algorithm [15,39], we identified regions in the human chromosome 1 containing tandem arrays of NBPF HORs. From these tandem arrays, individual NBPF monomers were extracted and classified into separate groups based on a 5% divergence threshold. This classification resulted in three distinct monomer groups, which we designated as m1, m2, and m3. A consensus NBPF monomer was then calculated for each group (Supplementary Table S1).

In the next step, these consensus monomers were utilized to scan the complete genomic sequences of humans, chimpanzees, gorillas, and orangutans for chromosome 1. For this analysis, we utilized the core tools integrated into our GRM2023 algorithm: MonFinder and GRMhor. MonFinder (available on github.com/gluncic/GRM2023, URL accessed on 11 December 2024) takes a genomic sequence (subject) and a consensus sequence (query) as the inputs, providing a list of detected monomers. The algorithm employs the Edlib open-source C/C++ library [40] for precise pairwise sequence alignment. Within MonFinder, the subject sequence is searched in both the forward and reverse complement directions to ensure the identification of all monomers. In this study, the consensus NBPF sequences obtained in the previous step were used as queries to detect all NBPF monomers in the genomic sequences under investigation. In the subsequent phase, the Python-based program GRMhor (available on github.com/gluncic/GRM2023) was invoked. It took as input a file containing the NBPF monomer sequences identified in the previous step. Upon processing the monomer array, GRMhor autonomously generated an MD diagram (see Section 3.4) and an aligned schematic representation of the monomer organization within the array (see Section 3.2 and Section 3.3).

## 3. Results and Discussion

### 3.1. GRM Diagrams

**Figure 1:**
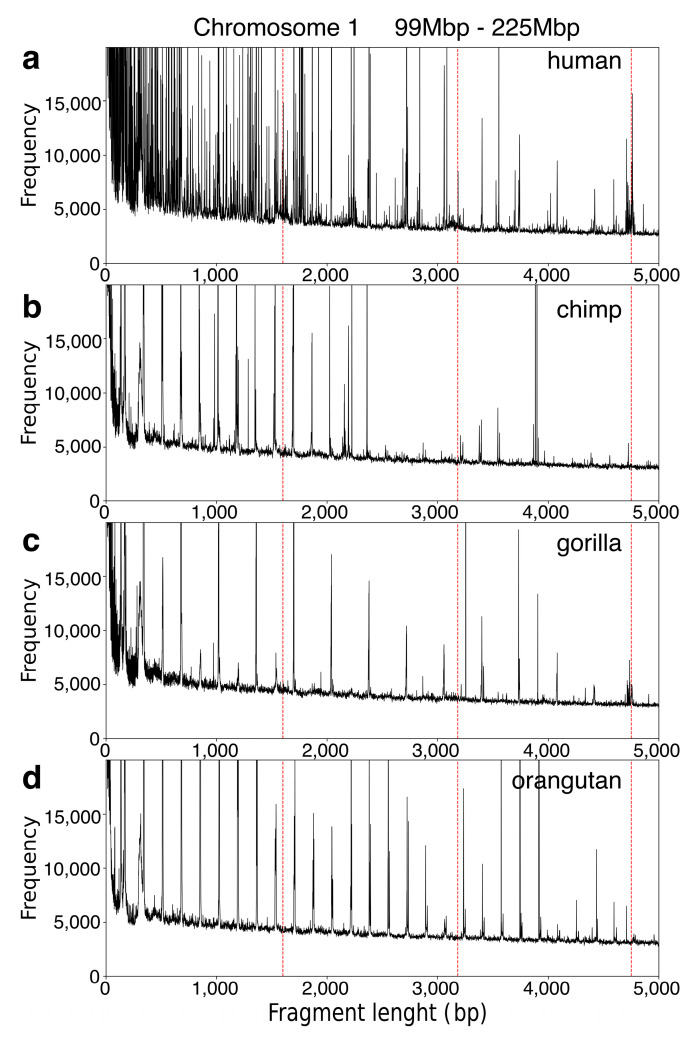
GRM diagrams for the 99Mbp–225Mbp segment of complete genomic sequences of higher primate RefSeq assemblies: (**a**) human, GCF_009914755.1 (T2T-CHM13v2.0); (**b**) chimpanzee, GCF_028858775.2 (NHGRI_mPanTro3-pri); (**c**) gorilla, GCF_029281585.2 (NHGRI_mGorGor1-v2.0_pri); and (**d**) orangutan GCF_028885655.2 (NHGRI_mPonAbe1-v2.0_pri). Dashed red lines indicate the positions of NBPF monomers (~1.6 kbp), as well as variant 2mer (~3.2 kbp) and canonical 3mer (~4.8 kbp) NBPF HORs. The GRM peak at ~4.8 kb corresponds to the NBPF 3mer HOR/Olduvai triplet. This peak is highly pronounced in the human genome, absent in chimpanzees and orangutans, and weakly present in the gorilla genome. These GRM diagrams highlight the strong human specificity of the NBPF 3mer HOR.

In the first step of applying the global repeat map (GRM) algorithm to the complete assemblies of human, chimpanzee, gorilla, and orangutan chromosome 1, we generated GRM diagrams for each primate genome (Figure 1a–d). Generally, peaks in the GRM diagram indicate the presence of repeat patterns, specifically corresponding to the *n*mer repeats as well as to intra- and inter-HOR copy repeats [13,41]. For human chromosome 1, the GRM diagram exhibits a prominent peak at ~4.8 kb, corresponding to the NBPF 3mer HOR/Olduvai triplet (Figure 1a). In contrast, the GRM diagrams for the chimpanzee and orangutan genomes lack a peak at ~4.8 kb, indicating the absence of the NBPF 3mer HOR/Olduvai triplet in the complete assemblies of their chromosome 1 (Figure 1b,d). The GRM diagram for the complete assembly of gorilla chromosome 1 reveals a weak peak at ~4.8 kb, suggesting a low-level presence of the NBPF 3mer HOR/Olduvai triplet.

The presented GRM diagrams (Figure 1) support the proposed methodology for identifying all NBPF monomers/Olduvai sequences across the analyzed species (see Materials and Methods Section). A pertinent question arises as to whether great ape genomes harbor additional NBPF monomers/Olduvai sequences that are significantly different (beyond the divergence threshold discussed in subsequent sections) from the human consensus sequences used for the search. The GRM diagrams clearly demonstrate that great ape genomes lack tandem arrays of NBPF monomers/Olduvai sequences, as well as canonical or variant NBPF HORs/Olduvai triplets composed of distinct monomers.

### 3.2. Aligned Monomeric HOR Schemes for NBPF 3mer HOR/Olduvai Triplet

**Figure 2:**
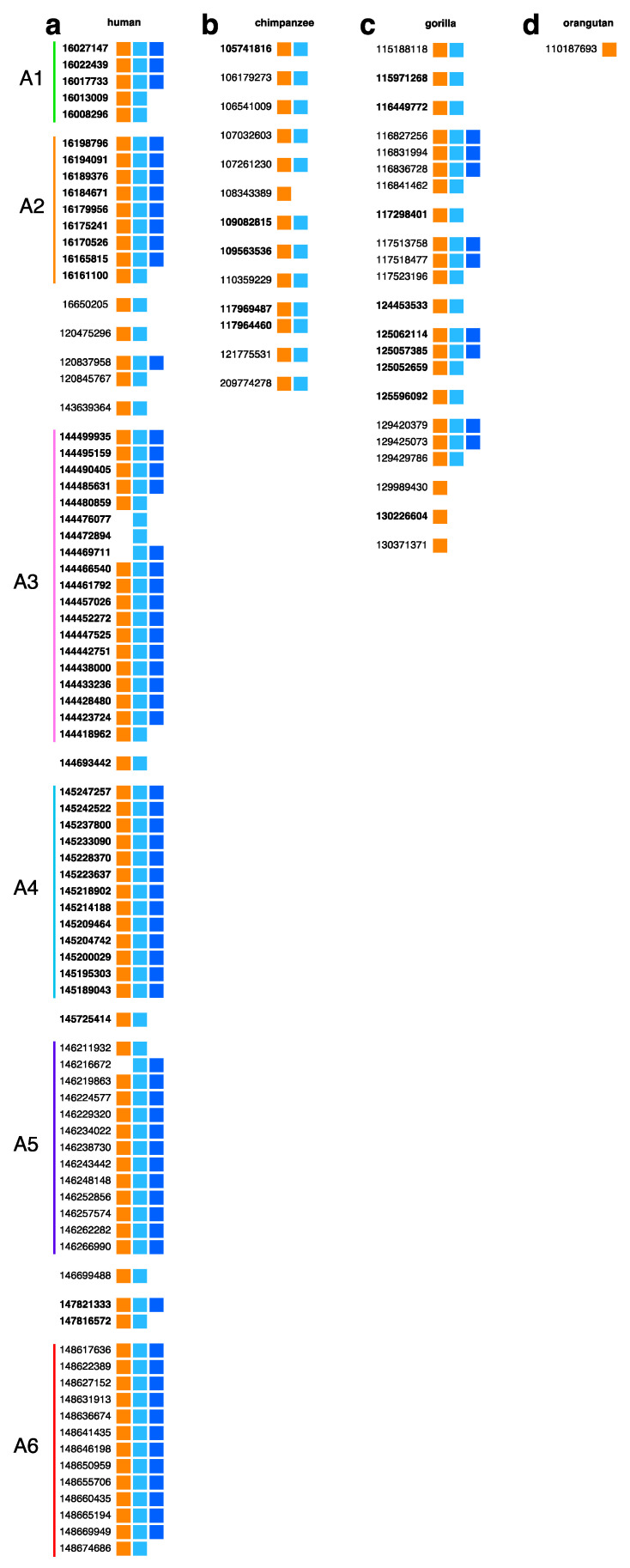
Alignment of the NBPF 3mer HOR/Olduvai triplet structure in human chromosome 1 and comparative NBPF monomeric patterns across great apes computed using a ≤5% divergence threshold from the human NBPF consensus monomers shown in the first panel. (**a**) Aligned monomeric scheme for NBPF 3mer HOR pattern in the human assembly GCF_ 009914755.1 (T2T-CHM13v2.0) (first panel). The computation was performed using the GRM algorithm. A 5% divergence threshold was applied to identify the three monomer types—m1, m2, and m3. Colored boxes represent the three NBPF monomer types: m1 (orange), m2 (light blue), and m3 (blue). According to the Sikela–van Roy terminology, these correspond to the HLS1, HLS2, and HLS3 repeat units, respectively [9]. Each row of boxes (three boxes in canonical HOR copies or two/one in variant HOR copies) represents an NBPF HOR copy. The starting genomic position of each HOR copy is shown before the row (bold for reverse complement). Arrays of tandemly arranged HOR copies are separated by blank spaces from neighboring arrays or individual HOR copies. (**b**) In chimpanzee assembly GCF_028858775.2, NBPF monomers were identified by their similarity to human monomers m1, m2, and m3, with a ≤5% divergence threshold. No complete NBPF HOR copies were detected in the chimpanzee genome. Instead, the genome contains variants: one tandem of two 2mer variants (m1, m2), ten individual 2mer variants (m1, m2), and one 1mer variant. (**c**) A similar search in the gorilla complete assembly GCF_029281585.2 revealed the presence of the following: one tandem comprising three canonical HOR copies (m1, m2, m3) and one 2mer variant (m1, m2); three tandems comprising two canonical HOR copies (m1, m2, m3) and one 2mer variant (m1, m2); six individual 2mer variants (m1, m2); and three 1mer variants (m1). (**d**) In the orangutan assembly GCF_028885655.2, only a single NBPF monomer was identified.

Using the GRM algorithm, the aligned monomeric scheme for the NBPF 3mer HOR pattern in the complete assembly of human chromosome 1 was computed and is presented in Figure 2a (first panel). The human assembly (first panel) contains six separated tandem arrays of HOR copies, denoted as A1–A6. Among these, tandem arrays A3, A4, A5, and A6 contain 20, 13, 13, and 13 HOR copies, respectively.

The tandem array A3 consists of 19 connected HOR copies with the following substructure: 4 canonical 3mer HOR copies, 4 variant HOR copies, 10 canonical HOR copies, and 1 variant HOR copy. In total, it contains 14 canonical and 5 variant HOR copies. The tandem array A4 includes 13 canonical HOR copies. Similarly, the tandem array A5 comprises 13 HOR copies, structured as 2 variant HOR copies followed by 11 canonical copies. Tandem array A6 consists of 13 HOR copies, with a substructure of connected 12 canonical and 1 variant HOR copy. These four tandem arrays (A3–A6) correspond to the NBPF genes NBPF20, NBPF19, NBPF10, and NBPF14, respectively.

The tandem arrays of NBPF 3mer HORs have been previously identified using the GRM algorithm. Specifically, NBPF20 was identified in contig NT_113799.1, NBPF19 in NT_079497.3, and NBPF10 in NT_004434 [13]. By applying the algorithm to detect the primary ~1.6 kb repeat units, the corresponding tandem arrays were also identified in the GRCh38 assembly, associated with genes NBPF20, NBPF19, NBPF10, and NBPF14 [7,22].

Additionally, two novel tandem arrays, designated A1 and A2, with five and nine HOR copies, respectively, were identified. These arrays correspond to two previously unknown NBPF genes, which were not present in the GRCh38 human genome assembly but were discovered in the complete T2T assembly of human chromosome 1. These novel genes have been named NBPFA1 and NBPFA2 [14].

In contrast, NBPF monomers in the chimpanzee assembly were identified by comparing the chimpanzee assembly to the consensus sequences of human monomers m1, m2, and m3, determined earlier using CVM computation for human chromosome 1. This analysis revealed that canonical NBPF HOR copies are entirely absent in chimpanzee chromosome 1 (Figure 2b, second panel). The same situation appears for the complete orangutan chromosome 1 (Figure 2d, fourth panel). However, in the gorilla genome, four short tandem arrays are present, comprising one array with four and three arrays with three NBPF HOR copies each. This weak exception in gorillas involves short NBPF tandems (with, at most, 4 tandemly organized copies), while the strong tandems in the human genome are in the range of 5 to 19 HOR copies.

This nonlinear amplification effect in the human genome might be attributed to the coordinated influence of tandemly organized NBPF 3mer HORs, which could interact synergistically to enhance their functional impact. In contrast, the isolated individual NBPF monomers, as seen in other primates, lack this structural organization and, therefore, might not produce a comparable cumulative effect. This hypothesis aligns with the observed correlation between the higher-order tandem organization of NBPF sequences and the advanced cognitive functions unique to humans, suggesting a significant evolutionary role for these genomic structures.

### 3.3. Dependence of NBPF Copy Number on Divergence Threshold for Monomer Type Classification

The NBPF copy number in a given sequence is influenced by the divergence threshold used to classify two monomers as belonging to the same type. A higher divergence threshold results in more monomers being grouped under the same type. Here, the concept of “monomer type” is defined here as a convenient framework for describing repeat structures, and it is inherently dependent on this arbitrary divergence threshold. For instance, with a divergence threshold of zero, only monomers with identical DNA sequences are classified as the same type. As the divergence threshold increases, more monomers are grouped into the same type, thereby altering the computed NBPF copy number for a given genomic sequence.

**Figure 3:**
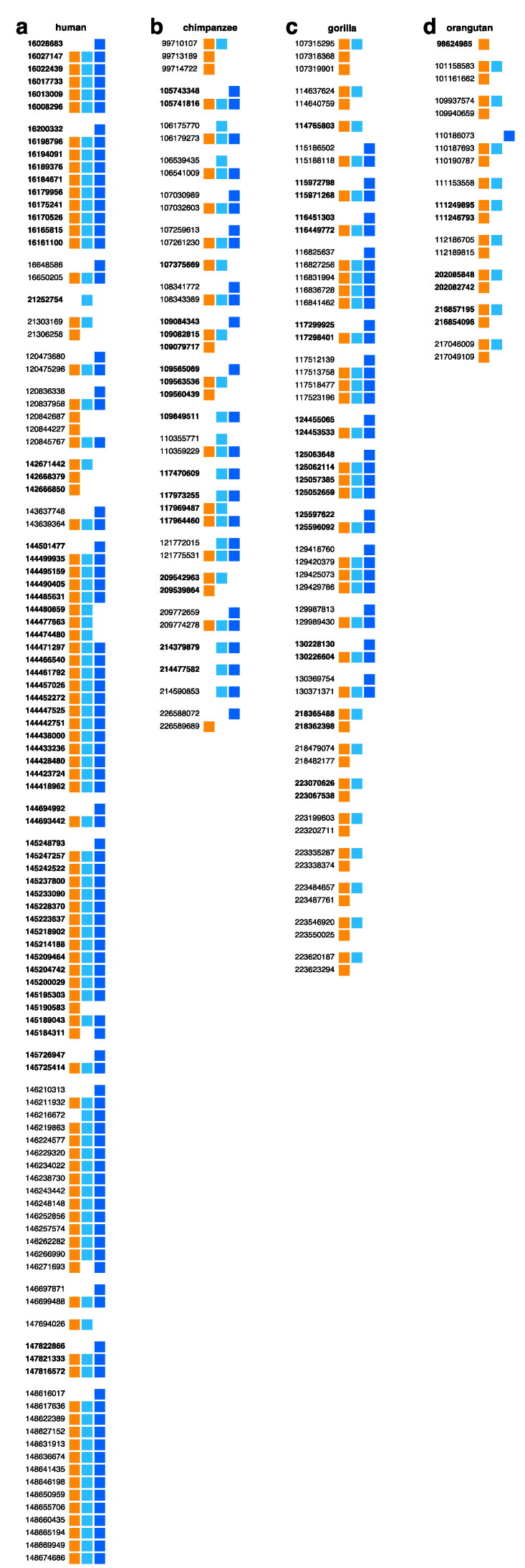
Aligned monomeric HOR scheme for the NBPF 3mer HOR/Olduvai triplet in the complete human chromosome 1, alongside the NBPF monomer schemes of great apes computed using a 20% divergence threshold from the human NBPF consensus monomers shown in the first panel. Colored boxes represent the three NBPF monomer types: m1 (orange), m2 (light blue), and m3 (blue). Each row of boxes (three boxes in canonical HOR copies or two/one in variant HOR copies) represents an NBPF HOR copy. The starting genomic position of each HOR copy is shown before the row (bold for reverse complement). Arrays of tandemly arranged HOR copies are separated by blank spaces from neighboring arrays or individual HOR copies. Aligned monomeric scheme for NBPF 3mer HOR pattern in the (**a**) human assembly GCF_009914755.1 (T2T-CHM13v2.0); (**b**) chimpanzee assembly GCF_028858775.2; (**c**) gorilla assembly GCF_029281585.2; and (**d**) orangutan assembly GCF_028885655.2.

In this study, we adopt a divergence threshold of 5% for human-aligned monomer HORs (Figure 2a). For comparison, we also present results using a 20% divergence threshold (Figure 3). Ideally, the chosen divergence threshold would reflect biological similarity in monomer activity within each type. Here, we align our choice with the 5% divergence threshold previously used by Willard and collaborators [11,42].

As shown in Figure 3, even with an increased threshold of 20%, the number of identified NBPF monomers/Olduvai sequences rises, yet a significant difference persists between humans and higher primates in NBPF higher-order structure-related patterns, particularly in the number of tandemly organized canonical 3mer HORs/Olduvai triplets. In humans, the number of tandem copies of 3mer NBPF HORs Olduvai triplets has increased to 68, while, in gorillas, it has risen to 13. However, in chimpanzees and orangutans, the number of tandem 3mer HOR copies remains zero even at this high threshold. Additionally, in humans, the structure comprising six HOR arrays corresponding to six NBPF genes remains intact.

We can conclude that, even at a very high threshold, the proportional difference in the number of tandem canonical HOR copies between humans and other primates remains largely unchanged. This further supports our hypothesis that the tandem organization of NBPF 3mer HORs/Olduvai triplets may significantly enhance cognitive capacity in humans compared to great apes, potentially providing a substantial evolutionary advantage.

There is experimental evidence from individual humans suggesting that certain cognitive functions increase with the number of Olduvai sequences [17,18,20]. However, this alone does not quantitatively account for the significant advantage of human higher cognitive functions compared to chimpanzees, given that humans only possess 2–3 times more Olduvai sequences than chimpanzees. Notably, the human genome contains approximately 50 tandemly organized Olduvai triplets/3mer NBPF HOR copies, which are completely absent in chimpanzees. This could provide a potential indication supporting the hypothesis that the tandem organization of NBPF 3mer HORs/Olduvai triplets contributes to enhanced cognitive functions. However, to our knowledge, there is currently no direct experimental evidence linking the tandem arrangement of 3mer HOR copies/Olduvai triplets to an additional enhancement of higher cognitive functions.

### 3.4. Monomer Distance (MD) Diagram

The MD diagram for a given genomic sequence represents the distance between the start of one NBPF monomer and the start of the next monomer of the same type, plotted against the enumeration of tandemly organized NBPF monomers. In general, an *n*mer HOR extending over an enumeration interval corresponds to a horizontal line segment in the MD diagram, located at the height of period *n* within that interval, analogous to the case of α satellite repeats, as described ref. [41]. In this study, the MD diagram method is extended to NBPF HORs.

**Figure 4:**
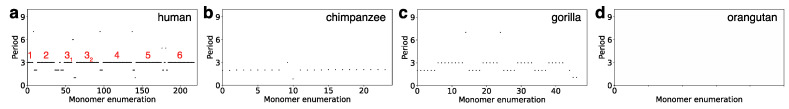
**Monomer distance (MD) diagrams for tandemly arranged NBPF monomers in the complete assembly of human and great apes’ chromosome 1**. The horizontal axis represents the enumeration of tandemly organized NBPF monomers, while the vertical axis indicates the period (distance between the start of one monomer and the start of the next monomer of the same type). Each point in the MD diagram corresponds to a monomer’s enumeration on the horizontal axis and its distance to the subsequent monomer of the same type along the sequence. These points form densely distributed horizontal line segments, which correspond to HORs. The vertical coordinate represents the period of the HOR [41]. (**a**) In the human MD diagram, seven horizontal lines can be observed at period 3, labeled 1, 2, $3_{1}$, $3_{2}$, 4, 5, and 6. The lines labeled 1 and 2 correspond to the HORs denoted as A1 and, respectively, at the top of Panel 1 in Figure 2. Similarly, the lines labeled $3_{1}\;and$ $3_{2}$ correspond to the first and second canonical segments of the tandem array labeled A3 in Figure 2, Panel 1. These two canonical segments belong to the same HOR tandem array with a variant 3mer HOR segment connecting them. The line segments $3_{1}\;and$ $3_{2}$ represent these two canonical sub-tandems, while a small blank space between them corresponds to four tandem variants that link them, forming the complete tandem array A3 depicted in Figure 2a. Finally, the line segments labeled 4, 5, and 6 correspond to the tandem arrays labeled A4, A5, and A6, respectively. (**b**) In the chimpanzee MD diagram, most points occur at period 2, indicating the presence of tandemly repeated 2mer variant HORs. (**c**) The gorilla MD diagram shows a periodic alternation of point clusters at period 2 and period 3, suggesting the presence of alternating tandemly repeated 3mer and 2mer variant HORs. The density of points is significantly lower compared to humans. (**d**) In the orangutan MD diagram, no points are observed, as the orangutan lacks any tandem arrays of NBPF monomers.

The MD diagram for the human genome (Figure 4) reveals six short horizontal line segments at period 3, corresponding to six NBPF genes. These segments are denoted in Figure 2 as subarrays A1–A6. Notably, the line segment corresponding to subarray A3, which consists of 19 tandemly connected HOR copies, is divided into two parts (denoted $3_{1}$ and $3_{2}$), separated by a blank space. This gap arises due to the presence of four variants inserted between two tandems of canonical 3mer HOR copies (see Figure 2a).

The MD diagram for the chimpanzee genome (Figure 4b) predominantly displays points at period 2, reflecting the tandem repetition of variant dimeric HORs (see Figure 2b). The MD diagram for the gorilla genome (Figure 4c) displays a slightly higher number of points at period 3 and period 2, although their density is significantly lower compared to the human MD diagram. An analysis of the origin of these points revealed the presence of one tandem consisting of three canonical 3mer HOR copies and one 2mer variant, as well as three tandems, each consisting of two canonical 3mer HOR copies and one 2mer variant (see Figure 2c). The MD diagram for the orangutan genome (Figure 4d) shows no points, as the orangutan lacks any tandem arrays of NBPF monomers (see Figure 2d).

### 3.5. Divergence Between Consensus Sequences of NBPF Monomers/Olduvai Sequences

**Figure 5:**
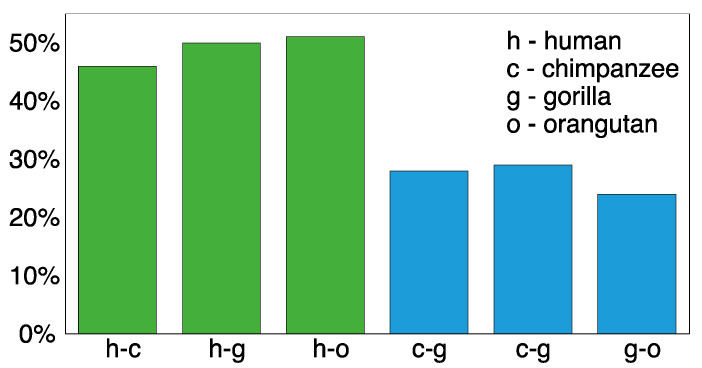
**Divergence between consensus sequences of NBPF monomers/Olduvai sequences in human and great apes.** A significantly higher divergence is evident between humans and great apes (first three green bars) compared to the divergence among different great ape species (last three blue bars).

The computed divergence values for human–chimpanzee (h-c), human–gorilla (h-g), human–orangutan (h-o), chimpanzee–gorilla (c-g), chimpanzee–orangutan (c-o), and gorilla–eorangutan (g-o) consensus monomers (Supplementary Tables S1–S4) are presented in Figure 5. These results reveal a significant difference, with the divergence between humans and great ape genomes being substantially larger (mean value: 49%) compared to the divergence among great apes (mean value: 24%).

In contrast, the divergence among monomers within each primate species varies. For m1/m2 divergence, the values are 20% in humans, 33% in chimpanzees, 21% in gorillas, and 29% in orangutans. Similarly, for m1/m3 divergence, the values are 19% in humans, 31% in chimpanzees, 26% in gorillas, and 30% in orangutans. These findings indicate a trend of increasing divergence of great ape genomes relative to the human genome, as well as notable differences in divergence among great ape species.

Analyzing complete genomes of primates provides valuable insights into the effect of divergence thresholds on the identification of NBPF monomers/Olduvai sequences. Although the absolute copy numbers of NBPF monomers/Olduvai sequences are dependent on the divergence threshold applied, the relative copy number patterns across different thresholds remain consistent. Notably, the divergence between human and great ape consensus sequences is significantly greater than that observed among great apes. Concurrently, humans exhibit a much higher number of tandemly organized canonical 3mer HOR copies/Olduvai triplets compared to the genomes of great apes. This distinctive organization suggests a possible link between the genomic architecture of these sequences and the cognitive and evolutionary advantages observed in humans. It is tempting to hypothesize that such a unique higher-order organization arises due to specific, yet unidentified, biological processes potentially tied to underlying genomic symmetries.

These findings align with and extend the current literature by emphasizing the potential evolutionary significance of tandemly organized Olduvai triplets in primates, particularly their association with cognitive capacity. Further investigation is required to elucidate the mechanisms driving these patterns and their functional implications.

## Figures and Tables

**Table 1:** Correspondence between Sikela–van Roy and Willard terminology in the description of NBPF repeats.

Sikela–Van Roy Terminology	Willard Terminology
Olduvai sequences (previously called DUF1220 sequences)	NBPF monomers
HLS1, HLS2, and HLS3	m1, m2, and m3
Olduvai triplets (previously called DUF1220 triplets)	NBPF 3mer HOR copies
NBPF genes	NBPF genes
Olduvai protein domain (previously called DUF1220 domain)	NBPF protein domain
CON1, CON2, and CON3	not included in 3mer HOR

## Data Availability

The MonFinder and GRMhor (Python applications) is freely available on github.com/gluncic/GRM2023. Reference genome sequences are freely available on the National Center for Biotechnology Information’s official website: T2T_CHM13v2.0 https://www.ncbi.nlm.nih.gov/datasets/genome/GCF_009914755.1/, NHGRI_mPanTro3-v2.0_pri https://www.ncbi.nlm.nih.gov/datasets/genome/GCF_028858775.2/, NHGRI_mGorGor1-v2.0_pri https://www.ncbi.nlm.nih.gov/datasets/genome/GCF_029281585.2/, and NHGRI_mPonAbe1-v2.0_pri https://www.ncbi.nlm.nih.gov/datasets/genome/GCF_028885655.2/ (all URLs accessed on 11 December 2024).

## Supplementary Materials

The following supporting information can be downloaded at https://www.mdpi.com/article/10.3390/genes15121598/s1: Table S1. Consensus canonical human NBPF monomers/Olduvai sequences; Table S2. Consensus canonical chimpanzee NBPF monomers/Olduvai sequences; Table S3. Consensus canonical gorilla NBPF monomers/Olduvai sequences; and Table S4. Consensus canonical orangutan NBPF monomers/Olduvai sequences.
