# Supplementary material for: Neuroblastoma Breakpoint Family 3mer Higher Order Repeats/Olduvai Triplet Pattern in the Complete Genome of Human and Nonhuman Primates and Relation to Cognitive Capacity
Source: Genes (Basel). 2024 Dec 13;15(12):1598. doi: 10.3390/genes15121598 (PMC11675761; doi:10.3390/genes15121598)
Supplement: Supplementary file 1 [file genes-15-01598-s001.zip › genes-3350816-supplementary.pdf]

Supplementary Materials for

NBPF (Neuroblastoma BreakPoint Family) 3mer HOR/Olduvai triplet pattern in complete genomes of higher primates (human, chimpanzee, gorilla, orangutan) and relation to cognitive capacity

Matko Glunčić, Ines Vlahović, Marija Rosandić, Vladimir Paar

Corresponding author: matko@phy.hr

The Supplementary file includes:
Tables S1 to S4

Table S1. Consensus canonical human NBPF monomers/Olduvai sequences.

Human m1
CCTGAAAGCTGGTCATGATATTCTTTGGTTTGCATCTCAGAACCAAGGGTGAAATATCCCTGTG
TGAGGATTCTGGTAGATCGTTATCCCAAAATCATTTATCCCAAGTTTGTGCAAACAGTTATGCC
TTATTGTTCCCATCAGTTCAAAGAAAATGCCCCAGATGATTTCTAGGAGGAAAACTGCAGTATT
CAGCCCTGTCTCATCAAATGCCCAGCTCGTTCATGGATGCAAGAATTTTAGACACTGAAATTAG
AATGAGGGAGGAAATCTACAAACCCTTGTGTCCAAATCATACTTCTGTGAATTTTTTACATCTG
CCTGGGTCCAATGTGCTGAGAGCGGGCTCAGGTTGCCACAGGCATGGCTGGAGACTAGGAATAG
AGCCTTGCTCACTGACCCATTTCATGTCTAGGCTTCCATGGAGACTACAGTTTCATTACAACCT
ATATGCGCCCATAGGTCCTGCCTGCGGCAATGACATCTCTCGGGTCAGTAAGGGCCACTTGGAA
CAGGAATATCACCCCTATCTGGAAGACCAGGTGGAGGCTTATCACCTTCACAGTAAGGTACTCA
CTGTCCACGTCAAGAGCCAAGCCAAGGTACTGTTCCTCCAATGAGTAAACAGCACTTTTGTAGG
GCTGGCCTAAGTCAGGCAGTTCAAGATAACCTGAAGGAGTCGAATAACATCTATCCAGTGAGTC
CTGCAAGACTTCAGGCTCTTTCTCATCCAGCAGCTCCCTGCTGAGCCTGGAAAAGTAGGAAAAA
GTAAAGAATAAGCCAGGGGGAATCAGAAACCACACAGCCCCAGCTACTTTTCATGGCTAACATA
AGGAACTGTTTAAAAAGAAAAAGGACAGATCCATTAATGAGGTAATGAATTATTGCCTTTATGT
TGGGATAGACCAGGGCCAGGTAGAAAAGAATGAAAGAGAAAGACAGGGAGAGGGAGAGAGAGAG
AGAGAGAGAGAGGAGAAAGTGAGCTCAGCGAATTGGCCGGGTGACACACTGATGAAGGGGTCAA
AGGACACTCTGAGTTAGTGCCCTCGGGACACACAGAGAACAGTGATCATGAAAAGAGTGGGCTC
AATAATTTTCCATAAACTTGCTCAAGATTCCATGCAGTTGCCATACAGCCTTTGAGGTATGGTC
AACCTATAGTAAGTTAGTAAATGATAAGGGGAGGAAGAAATGGAAACCTAAACATCTACTGCAA
TGAAAACCAACAGCAATGTCAGTAGGAGTAATTCAACCTTCGTTGAAAACATGAAATTGAACAC
ACTCTTGTTTTCCCTGGACCTGGCATCTCCAGGTGTCAACACAGAATTAAGCATCCATAATTGC
TCAAAGTTACCTGGGGCATGATGGGTCTTGGTCTTCTTCCACTTCTTGGTACTTTTCAATTTCT
GCAATAAGTTCAGACATGGACAGACATATTAAGCTGGTTCTCCTACACACATAACAATCCACTG
TCTAATCCTCACACAGGGACTTCAGGCTCCTCAGCATGAGAATAGGACACTGTGAGAGATAGTC
TTCAGGAGGCTTGAAGG

Human m2
CCTGAAGGCTGGTCATGATAGAAATTCCTCGGTTTTTCTCCCAGAAACTGTGGGTAAAATGTCC
CTATTCTAGTAGATCGTTATCCCAATATCATTTGTCCCAAGTTTGTGCAAACAGTTATGCCATA
TTTTTCCAATCAACTTAAAGCAAATACCCTCAAATGATTTCTAGGAGAAAAACTGCAATATTTA
GCCCTGTCTCATCAAATACTCAGATTGTTCATGGTTGTGAGGACTTTAGACACTGAAATTAGAG
TGAAAAAGGAAATCTACAAACCCTTGAGTCAAAATCATAGTTCTCTGAATTTGTCACATCTGCC
CAGGTCCAATGTCATGAGAGTAGATATCAGGGCGCCACAGGTATGGCCTGAGACTAGGAAGAGA
GTCATGCTCACTGACCCATCCCTTGTCTGGGCTTCCAGGTAGAACTAGAGTTTCATTCAACCTA
CATGTGCCTATAGGTCCTCCCTGCACAATGACATCTCTCAGCTCAGTAATGGCCACTTGGAGCA
GGAATATGATCTTTATATGGAAGACTCAGTGGATCCTTATCACCTTCATAGAAAGGTACTCACC
TCCCACGTCAAGAGAAAAGCCAACATGTTTTTCCTCCAATGCATAAAAGGAACTTCCATAGGGC
TGGCAGGAGTCAGGCTGTTCAAGACAACTGGAAGGAGTTGAATAACATCTATCCAGTGAGTCCT
GCAAGACTTCAGGCTCTACTGCCTCCAGCAGCTCCCTGCTGAGCCTGGAAAAGGAGGAAAAAGT
AAAGAATAAGCCAGGGGAAATCAGACACAACAGAGCCCCAACTAGGTTTCATGGGTAGCATAGA
GGAAGTGGTTGCAAAACTAAAAGGATAGATCCATTAATGAGGTAACAAATTATTGCCTTCATGT
TGGGACAGAACAGGGCCAAATGGAAAAGAATGAAAGAGAAAGACAGATAGACACACACACACAC
ACACACACACACACACACACACACACACACACACACACACACACAGAGAGAGAGAACGAGCTCA
GTGAATTGTCCAGGTGACACACTGATGAGGGAGTAACAGGACACTCTGAGTTAGTGCCCTCAGG
ACACACAGCATACAGTGATCAGGAAAAGACTGTGCTCAATAATTTTCCATAAAATGTGCTCAAG

TTTCCATGCAGTCGCCATGAGAATACAGTTTTTGAAGTCTGGTCCACCTACAGTAGGTTAGTAA
ATGATAAGGGGAGGAAGAAATGGAAACCTAAATATCTACTGCAATGAAAACCAACAGCAATGTT
AGTAGGAATAATTCAGGCTCATGACATTGAAAAGATGTAATCGATAATGTCAGCCCGCTATGTT
TTCCCTGAACCAGGAGTCTCCAGATGTCAACACAGAAGTAGCTGTTCACAATTGCTCAGTTACC
TGGGGCATGGTGGGCCTTGGTCTTCTTCCTCTTCTTGGTCCTTTTTAATTCCTGCAATACATTC
AGACAGGGACAGACAAAATAAGCCAATTCACCTACACCCATAACAGTCCACTGTCTAATCCCCA
CACAGGGATCTCAGGCTCCTCAGCATGAGAACAGGACAATGTGAGAGATATACTTCAGGAGG

Human m3
CCTGAAGGCTGATCACCATAGAGATTCCTTGGTTTTTGTCCCAGAAACTGTGGGTAAAATTCCC
TATTCTGGTAGATCGTTATCCCAATATCATTTGTCCCAAGTTTGTGCAAATGGTTATGCCATAT
TTTTCCAATCGATTTAAAGCAAATGCCCCCAAATGGTTGCTAGGAGAAAAACTGCAAGATTCAG
CCCTGTCTCATCAAATACTCAGATTGTTCAGGGTAGCGAGGATTTTAGACGCTGAAATTAGAGT
GAAGGATGAAATCTACAAGATCTACAAAATTGAGACAAAATCAGAGTTGTGTGAATTTGTCACA
TCTGCCCAGGCAACAGATTGAGAGTAGGATTAGGGCGCCACAGGCATGGCCTGAGACTAGGAAG
AGAGCCTTGCTCACTGACCCATCCCTTGTCTGGGCTTCCAAGTGGAACTAGAGTTTCATTCAAC
CTACATGTGCCTATAGGTCCTCCCTGTGGCAATGACATCTCTCAGCTCAGTAAGGGCCACTTGC
AGTAGGAATATGACCCTAACCAGAAGACTCAGTGGATCCTTATCACCTTCATAGAAAGGTACTC
ACCATCCATGTCAAGGGCCAAGCCAACACGCTGTTGCTCCAATACGTAAAAGGCACTTCTGTAG
GGCTGGCATGAGTCAGTCAGTTCAAGACAACCTGAAGGAGTTGAATAACATCTATCCAGTGAGT
CCTGCAAGACTTCAGGCCCTTTCTCATCCATTGTCCCTGCTGAGCCTGGAAAAGTGGAAAAAG
TAAAGAATAAGCCAGGGGGAATCAGAAACCACACAGCCCCAGCTAGATTTCATGGCTAACTTAA
GGAAGAGTTTGAAAAGAAAAAGGACAGATCCATTAATGAGGTAACAAATTATTGCCTTTATGTT
GGGATAGAACAGGGCCAGGTAGAAAACAATGAAAGAGAAAGACTCACTGGACAGAGACAGAGAC
AGAGACAGAGACAGAGACAGAGAGAAAGTGAACTAGTGAATTGGCCAGGTGACATACTGGTAAG
GGAGTAAAAGGACACTCTGAGTTAGTGCCCTCATGACACACAGCAAACTGTGATCATGAAAAGA
GTGAGCTCAATAGTTTTCCATAAAATATGCTCAAAATTCCTACTGCAGTGGCCATGAGAGTACA
GCTTTTGAAGTATGGTCATTATGGTACGTTAGGAAATGATAAGGGGAGGAAGAAATGGAAACCT
AAACATCTACTGCAATGAAAACCAACAGCAATGACAGTAGGAGTAATTCAGCCTTCGTTGAAAA
CATGCAAACACACTCTGGTTTCCCTGAATCTGTTGCCTCCAGGTGTTAACACAGAATTAAGCAT
CCACAATTGCTGAAAGTCACCTGGGGCATGGTGGGTTTTGATCTTCTTCCCCTTCTTTTCTTCC
CCTTACCATTCTTTGAGTATTCTTTGATCTTCTTCCCCTTCTTTTCTTCCCCTTCCCCTTCTTT
TCAATTTCTGCAATAAATTCAGACATGGACAGACACATTAAGCTGATTCCCCTACACACATAAC
AATCCACTGTCTAATCCTCACACAGGGACCTCAGGCTCCTCAGCATAAGAATAGGACACTTTGA
GAGATATATTTCAGGAGGCCTGAAGCCTTCAGT

Table S2. Consensus canonical chimpanzee NBPF monomers/Olduvai sequences.

Chimpanzee m1
CAGCCTTCAGGCCTCCTGAAGAATATCTCTCACAGTGTCCTATTCTCATGCTGAGGAGCCTGAA
GTCCCTGTGTGAGGATTAGACAGTGGATTGTTATGTGTGTAGGAGAACCAGCTTAATATGTCTG
TCCATGTCTGAACTTATTGCAGAAATTGAAAAGTACCAAGAAGTGGAAGAAGACCAGTACCCAT
CATGCCCCAGGTAACTTTGAGCAATTTCATGGATGCTTCATTCTGTGTTGACACCTGGAGATGA
ATCCAGGGAAAACAAGAGTGTGTTCATTTCATGAATTTTCAACGAAGGTTGAATGGGTCCTACT
GACATTGCTGTTGGTTTTCATTGCAGTAGATGTTTAGGTTTCCATTTCTTCCTCCCCTTATCAT
TTACTAACTTACTGTAGGTTGACCATACCTCAAAGGCTGTACCCATTTCATGTCTGCATGGAAT
CTTCAAACTTATGGAAAATTTTGAGCCAACTCTTTTCATGATCACTGTTCGCTGTGTGTCCTGA
GGGCACTATGACAGAGTGTCTCTTGACCCCTTCATCAGTGTGTCACCCGGCCAATATCACTGAG
CTCACTTTCTCCTCTCTCTCTCTCTTATCACCTTCATCCCTCTCCCTCACTGTTCTCTTTCATT
CTTTTCTACCTGGCCCTGTTCTATCCCAACATGAAGGCAATAATTCATTACCTCATTAATGGAT
CTGGCCTTTTTCAAACAGTTCCTTATGTTAGCCATGAAATCTAGCTGGGGCTCTGTGGTTTCTG
ATTCCTGGCTTATTCTTTACTTTTTTCTACTTTTCCAGGCTCAGCAGGGAGCTGCTGGAAAAGA
AAGAAAAAGCCTGAAGTCTTGCAGGACTCACTGGATAGATGTTATTCACACTCCTTCCAGTTAT
CTTGAACTGCCTGACTCATGGCCAGCATACAGAAGTGCTGTTTACTCATTGGAGGAACAGGACC
TTGGCTTGGCTCTTGATGTGGACAATGAGTACCTTACTATGATGGTGATAAGGATCCACCTGGT
CTTCCAGATAGAAAAGTGATATTCCTGTTCCAAGTGGCCCTTACTGACCCGAGAGATGTCATTG
CTCAGGCAGGACCTATGGGTGCACTGATGAAGGTTGTAATGAAACTCTAGTTAGTGCCCTGGAA
GCCTAGACATGAACATGGGTCAGTGAAAAGCAAGGCTCTATTCCTAAACTTCTCAAGATTCCAT
GCCAGTGGCAACCTGAGCCAACCTACAGTCTTAGTACATTGGACCCAGGAAGAAATGTAAAACC
TTCACATCTACTATGAAAACCAACAGCACTGTCAGTAGGAGTAGATCTCCTTCATTCTAAGTGT
CTAAAATGAAATTGCAACCATGAACGAGCTGGGCATTTCCAGGTGTCAACAGGGCTGAATACTG
CAGTTTTCCTCCTAGAAATCATCTGGGGCATGATTCTTTGAACTGATGGGAACAATAAGGCATG
CTGTAAGCACAAACTTGGGATAAATGATGGTTTGGGATAACAATCCACCTGTCTAATCCTCACA
CAGGGACTTCAGGCTCCTTGGTTCTGAGAATAGGACACTGTGAGAGAATATCATGACCAGCCTT
CAGG

Chimpanzee m2
CCTTCAGGCTGGTCCTGATAGTAAATTCCTCACATTTTCTCCCAGAAACTGTGGGTAAAATCCC
TCTAGTAGATCGTTATCCAGTGGAATATTATTTGTCCCAAGTTTGTGCAAACAGTTATGCCTTA
TTTTGTCTGTCCAATCAATTTATTGCAGCAAATAAAAATGATTTCAAGAAGAGAAGAACTGCAA
GGCCCATTTAGCCCTGTCTCATCAATTACTCAGATTACTTCATGGTTGTGGAGACTTTGGACAG
GGAAATTAGAGTGAAAAAGGAAATCTACATCTTGAGTCAATCATAGTTCTCTGAATTATTCCTA
CTAACATCTGCTGTCCAATGTCATGAGAGTAGATATTTAGTGCCACAGGCATGGCCTGAGACTA
GGAAAGCCATTCTCACTGACTGCATGGAATGTCTGGGCTTCCAGGGAGAACTAGAGCACAGTCT
TTTCATGATCAACCTATGCTGTGTGCCTGAGGTCACTCACTGCAGCAATGTCCTGTCTCAGCTC
ATCAGTGTGTCACTTGGACAGGAATCATGAGCTCGTTCTCTCTCTTTATATGGAAGACTCAGTG
GTCCTCTATCACCTTCATTTGGTACTCACCTCCCACGTCAAGAGAAAAGCCAACATGTTTGTTA
CCTCATTAATGGATCTTCCTTAGGCTTTTTAAGGAGTTCCTTAGGCTACCCATGAAAACTAGA
AGGGGCTTGTTGTAACTGATTATCCCTGGCTTATTCTTTACTTTTTCCTACTTTTCCAGGCTCA
GCAGGGAGCTGCTGGAAGTAGAAAGAGCCTGAAGTCTTGCAGGACTCACTGGATAGATGTTATT
CAACTCCTTCCAGTTGTCTTGAACAGCCTGACTCCTGCCAGGCCTATGGAAGTTCATTTTATGC
ATTGGAGGAAAAACATGTTGGCTTTTCTCTTGACGTGGGAGGTGAGTACCTTTCTATGAAGGTG
ATAAGGATCCACTGAGTCTTCCATATAAAGATCATATTCCTGCTCCAAGTGGCCATTACTGAGC
TGAGAGATGTCATTGCTGCAGTGAGGACCTATAGGCACATGTAGGTTGAATGAAACTCTAGTTC

TACCTGGAAGCCCAGACACATGGGTCAGTGAGCATGGCTCTCTTCCTAGTCTCAGGCCATACCT
GTGGCACTCTGATTCTACTCTCATGACATTGGACCTGGGCAGATGTGACAAATTCAGAGAACTA
TGATTTTGACTCAAGGAAGTTTGTAGATTTCCTTTTTCACTCTAATTTCAGTGTCTAAAGTCCT
CACAACCATGAACAATCTGAGTATTTGATGAGACAGGGCTAAATATTGCAGTTTTTCTCCTAGA
AATCATTTGAGGGTATTTGCTTTAAATTGATTGGAAAAATATGGCATAACTGTTTGCACAAACT
TGGGACAAATGATATTGGGATAACGATCTACTAGAATAGGGACATTTTACCCACAGTTTCTGGG
AGAAAAACCACACAGGAATTTCTATCATGACCAGCCTTCAGGACAATGTGAGAGATATACTTCA
GGAGG

Chimpanzee m3
GCCTTGCCCAAGGCTCTAATGAAAGAACTCAAGCCAGTTTTCTCAAGCCTTGTCAGGCCTCCTG
AAATATATCTCTCAAAGTGTCCTATTCTTATGCTGAGGAGCCTGAGGTCCCTGTGTGAGGATTA
GACAGTGGATTGTTATGTGTGTAGGGGAATCAGCTTAATGTGTCTGTCCATGTCTGAAGGTATT
GCAGAAATTGAAAATGAAGGGGAAGGGGAAGAAAAGAAGGGGAAGAAGATCAAAACCCACCATG
CCCCAGGTAACTTTCAGCAATTGTGGATGCTTAATTCTGTGTTAACACCTGGAGGCAACAGATT
CAGGGAAAGCAGAGTGTGTTTGATGGCATGTTTTCAACGAAGGCTGAATTACTCCTACTGTCAT
TGCTGTTGGTTTTCATTGCAGTAGATGTTTAGGTTTCCATTTCTTCCTCCCCTTATCATTTCCT
AACGTACCATGCAGGTTGACCATACTTCAAAAGCTGTACTCTCATGGCCACTGCATCGAAATTT
GAGCATATTTTATGGAAAACTATTGAGCTTGTCACTCTTTTCATGATCACCGTTTGCTGTGTGT
CATGAGGGCACTAACTCAGAGTGTCCTTTTACTCCCTTACCAGTAAAACACACCTGGCCAATTC
ACTAGCTCACTTTGACTCTGTCTCTGTCCAGTGAGTCCTGTCTTCTGTCTTTCTCTTTCATTGT
TTTCTACCTGGCCCTGTTCTATCCCAACATAAAGGCAATAAGCCTCATTAATGGATCTATCCTT
TTTCTTTTCTAACACTTCCTTATGTTAGTTCTGAAATCTAGTGGGGCTCCATTAATGTGGTGTC
TGATTTTCCCTGGCTGCTTCTTTAGTTTTGTCTCCTTTTCCAGGCTCAAGGGCCTGATGGAAGT
GAAAGAGCCTGAAGTCTTACAGGACTCACTGGATAGATGTTATTCGACTCCGTCAATGTACTTT
GAGCTACCTGACTCATTCCAGCACTACAGAAGTGTGTTTTACTCATTTGAGTAAAAGCACACTC
TGAGCTTCGCCCTTTACATGGACACATAGGTTTTTTACTTTGATCATGAAAAGTGAGCTCCACC
TAGTTTTCCATAAAATATTCCCACAATTCGATGCCCTTACTATGAGAGATGTCATTCCTGAGGT
AGGACCTATAGGCACGTGAAGATTTGAATGAAACTCTAGTTCCATTTGGAAGCCCAGACATAGG
ATGGGTCAGTGGGCATGGCTCTAGCCTATTCTCAAACCATGCCATCAAACCTGTGCTCAGTCTG
AAGATCTTGGACCCTCCAGGTGTGACACATTCACATTAACTGTGCAGCAATTGCTCAAAGTTAC
CTGGGACATTTTAATTTGATCCTCTCTTAGCTACAAAATTCCTCAGGGATTTCATTTTGCAGGC
ATTCTCTGATGAGACCCTGCTCAAGGTCAGTTCATCCTTGTGTTTAGCTCATCCAAAATTTCTG
GTTTCAATAAATCCTAACTCAGTGTCATCAGTGTTGGCTTGTTTTAGCTGATCCATCTGTAACA
CAGGAGGGATCCTTGGCTGAGGATAATCCTCACACAGGGACCTCCCTCAGCTCTTGACAATTGT
TAACCCGACACTGTGGATATCTTTCAGGAGGCCTGAAG

Table S3. Consensus canonical gorilla NBPF monomers/Olduvai sequences.

Gorilla m1
CCTTCAGGCCTCCTGAAGAATATCTCTCACAGTGTCCTATTCTCATGCTGAGGAGCCTGATATC
CCTGTGTGAGGATTAGACAGTGGATTGTTATGTGTGTAGGAGAACCAGCTTAATGTGTCTGTCC
ATGTCTGAACTTATTGCAGAAATTGAAAAGTACCAAGAAGTGGAAGAAGACCAAATGCCCATCA
TGCCCCAGGTAACTTTGAGCAATTATGGATGCTTAATTCTGAACCTGGAGATGCCAGGTCCAGG
GAAAACAACCCTTCAATTTCATGTTTTCAACGAAGGTTGAATTACTCCTACTGACATTGCTGTT
GGTTTTCATTGCAGTAGATGTTTAGGTTTCCATTTCTTCCTCCCCTTATCATTTACTAACTTAC
TGAGCCATGTTGACCATACCTCAAAAGTCTGTATGGCAACTGCATGGAATCATGAGCAAGTTTA
TGGAAAATTATTGAGCCCACTCTTTTCATGATCACTGTTCAGTGTGTCAGTAAGGGCACTAACT
CAGAGTGTCCTTTGACCCCTATCATCAGTGTGTCACCTGGCCAATTCACTGAGCTCACTTTCTC
TCTCTATCTCTCTCTCTCTCTCTGTCCCTCTCAAGAGCCAAGCCAAGGTCTGTCTTTCTCTTTC
ATTGAGTTCTACCTGGCCCTGGTCTATCCCAACATAAAGGCAATAATTTGTTACCTCATTAATG
GATCTATCCTTTTTCTTTTTAAACAGTTCCTTATGTTAGCCATGAAATCTAGCTGGGGCTGTGT
GGTTTCTGATTCCACCCTGGCTTATTCTTTACTTTTTCCTACTTTTCCAGGCTCAGCAGGGAGC
TGCTGGAAGCAGAAAGAGCCTGAAGTCTTGCAGGACTCACTGGATAGATGTTATTCGACTCCTT
CAGGTTATCTTGAACTGCCTGACTTAGGCCAGCCCTACAGAAGTGCTGTTTACTCATTGGAGGA
ACAGTACCTTGGCTTGGCTCTTGACGTGGACAGTGAGTACCTTACTATGAAGGTGATAAGGCTC
CACCTGGTCTTCCAGATAGGACATATTCCTGTTCCAAGTGGCCCTTACTGACCCGAGAGATGTC
ATTGCCGCAGGCAGGACCTATGGGCGCATATAGGTTGTAATGAAACTGTAGTTTCAGTTGGAAG
CCTAGACATGAAATGGGTCAGTGAGCAAGGCTCTATTCCTAGTCTCCAGCCATGCCTGTGGCAA
CCTGAGCCCAGTCTCAGCACATTGGACCCAGGCAGATGTAAAAAATTCACAGAACTATGATTTG
GACACTCAAGGGTTTGTAGATTTCCTCCTTCATTCTAATTTCAGTGTCTAAAATTCTTGCAACC
ATGAACGAGCTGGGCATTTGATGAGACAGGGCTGAATACTGCAGTTTGTCTCTTAGAAATTCTT
GGTCTGGGTCATTTTCTTTGAATTGATGGGAACAATAAGGCATAACTGTTTGCACAAACTTGGG
ATAAATGATTTTGGGATAACGATCTACCAGAATAGGGACTTCAGGCTCCTCAGGTTCTGAGATG
CTGTGAAGAATATCATGACCAGCTTTCAGG

Gorilla m2
CCTCCTGAAGTATATCTCTCACATTGTCCTGTTCTCATGCTGAGGAGCCTGAGATCCCTGTGTG
GGGATTAGACAGTGGACTGTTATGGGTGTAGGTGAATTGGCTTATTTTGTCTGTCCCTGTCTGA
ATGTATTGCAGGAATTAAAAAGGACCAAGAAGAGGAAGAAGACCAAGGCCCACCATGCCCCAGG
TAACTGCAATATTGAGCAATTGTGAACAGCTACTTCTGTGTTGACATCTGGAGACTCCTGGTTC
AGGGAAAACAGAGCGGGCTGACATTATCGATTACATCTTTTCAACCAAGCCTGAATCATTCCTA
CTAACATTGCTGTTGGTTTTCATTGCAGTAGATATTTAGGTTTCCATTTCTTCCTCCCCTTATC
ATTTACTAACCTACTGTAGGTGGACCATGCTTCAAAAACTGTATTCTCATGGCGACTGCATGGA
AACTTGAGCACATTTTATGGAAAATTATTGAGCACAGTCTTTTCATGATCACTGTATGCTGTGT
GTCCTGAGGGCACTAACTCAGAGTGTCCTGTTACTCCCTCATCAGTGTGTCACCTGGACAATTC
ACTGAGCTCGTCTCTCTCTCTGTGTGTGGAATGTGTGTGTGTGTGTGTGTGTGTGTGTGTGTGT
GTCTGTCTGGAAGACTCAGTGGATCCTTTCACCTTCATTCTTTTCCCTGTTCACGTCCCAACAT
GAAGGCAATAATTTGTTACCTCATTAATGGATCTATCCTTTTACTTTTTTAACCACTTCCTTAT
GCTACCCATGAAACCTAGTTGGGGCTCTGTTGTGTCTGATTTCCCCTGGCTTATTCTTTACTTT
TTCTACTCCTTCCAGGCTCAGCAGGGAGCTGCTGGAGGTAGTAGAGCCTGAAGAATCTTGCAGG
ACTCACTGGATAGATGTTATTCAACTCCTTCCAGTTGTCTTGAACAGCCTGACTCATGCCAGCC
CTATGGAAGTTCCTTTTATGCATTGGAGGAAGAACATGTTGGCTTTTCTCTTGACGTGGGAGGT
GAGTACCTTTCTATGAAGGTGATAAGGATCCACTGAGTCTTCCATATAAAGATCATATTCCTGC
TCCAAGTGGCCATTACTGAGCTGAGAGATGTCATTGCTGCAGTGAGGACCTATAGGCACATGTA

GGTTGAATGAAACTCTAGTTCTAATTGGAAGCCCAGACATGGGATGGGTCAGTGAGCATGGCTC
TCTTCCTAGTCTCAGGCCATGCCTGTGGCACTCTGATTCTACTCTCATGACATTGGACCTGGGC
AGATGTGACAAATTCAGAGAACTATGATTTTGACTCAAGGGTTTGTAGATTTCCTTTTTCACTC
TAATTTCAGTGTCTAAAGTCCTCACAACCATGAACAATCTGAGTATTTGATGAGACAGGGCTGT
TGCAGTTTTTCTCCTAGAAATCATTTGAGGGCCTTTGCTTTAAATTGATTGGAAAAATATGGCA
TAACTGTTTGCACAAACTTGGGACAAATGATATTGGGATAACGATCTACTAGAATAGGGACATT
TTACCCACAGTTTCTGGGAGAAAAACCACACAGGAATCTCTATCATGACCAGCCTTCAGACAAT
GTGAGAGATATACTTCAGGAGG

Gorilla m3
AGCCTTCAAGGCTCCTGAAATAGAGTTTCCTATTCTTGTCCTGAGGAGCCTGAGGTCCCTGTGT
GAGGATTAGACAGTGGATTGTTATGTGTGTAAGGGAATCAGCTTAATGTGTCTGTCCAAGTCTG
AATTTATTGCAGAAATTGAAGGATAGGGGAAAAAGAAGGGGAAGAAGATCAAAGAAGAAAAGAA
GAAGGGGAAGAAAAGAAGGGGAAGATCGATCAAAACCCAAATGCCCCAGGTGACTTTCAGCAAT
TGTGGATGCTTAATTCTGCCCTGTTAACACCTGGAGGCAACAGTTCAGGGAAACCAGAGATGTG
TTTGATGTCAAATTAACGAAGGCTGAATTACTCCTACTGTCATTGCTGTTGGTTTTCATTGCAG
TAGATGTTTAGGTTTCCATTTCTTCCTCCCCTTATCATTTACTAACATACCATAGGTTGACCAT
ACCTCAAAAGCTGTACCCTCATGGCCACTGCTTCCAATTTTGAGCATATTTTATGGAAAACTAT
TGAGGTCACTCTTTTCATGATCACATCTCTGTGTGTCATGAGGGCACTTGCTCAGAGTGTCCTT
TGACTCCCTCCAGAAGACCAGTGGATCCTTGTCACCTGGCATATTCACTAGCTCACTTTCCATG
TCAAGGGCGAAGCTCTGTCTCTGTTGCTCTGTCTAAAAGGCACTGTCTGTCTTTCTCTTTCATT
GTTTTCTACCTGGCCCTGTTCTATCCCAACATAAAGGCAATAAGTTACCTCATTCATGGATCTT
CCTTTTTCTTTTCAAGCTCTTCCTTATGTTAGCCATGAAATCTAGCTGGGGCTGCCAGTGGTTT
CTGATTCCCCCTGGCTTATTCTTTAGCCCCACTTTTCCAGGCTCAACAAGGAAGAGTTGGAAAA
GAAAGAGCCTGAAGTCTTAAGGACTCACTGGATAGATGTTATTCAACTCCTTCATGTTGGGATT
GAACTGACTGACTCATGCCAGCCCTACAGAAGTGCCAGGTTACATATTGGAGGGAGTAACAGCG
TGTCTGAGCTTGGCTGTTGACATGGACACACAGCTTTCTATGATCATGAAAAGGATCCAATGAG
TTTTCCATAAAATATTCCTACTGCAAGTGGCCCTTACTGAGCTAGAGATGTCATTGAAGTAGGG
TCAACCTATAGGCACATGTAGTGAATGAAAGGGGAGTTCCAAATGGAAGCCCAGACATCTACTG
GGTCAGTGAAAACCAACAGCAAGGCTCTAGGCCTAATCTCAGGCCATGCCTGTGGCAACCTAAT
CAAACACACTCTGGTTTCCCTGAAGATGTTGGATCTCCAGGTGTGACAAATTCACACAACTTGC
AGTACCTGGGGCTTGTAGATTTCATCCTTCACTCTAATTTCAGCGTCTAAAATCCTCGCTACCT
GAACAATCTGAGTATTTCATGAGACAGGGCTGAATCCCCAGTTTTTCTTCCCTAGCAACCATTC
TGGGGGCATTTTTGCAATAAATCGATTGGAAAAATATGGCATTACCATTTGCACAAACTTAACA
ATGATATTGGGATAACGATCTACCAGAATAGGGAATTTTACCTCCTCACAGTTTCTGGGACACT
GGCTGAGGAATCTCTTTCAGGATCAGCCTTCAGG

Table S4. Consensus canonical orangutan NBPF monomers/Olduvai sequences..

Orangutan m1
CCTTCAGGCCTCCTGGTTTGCATCTATCTCTCAAGGGTCCTATTCTTATGCTGAGGAGCCTGAT
CCCTGTGTTTGGATTAGACAGTGGATTGTGCAAACAGTGTGTAGGGGAATCAAGATGTGTCTGT
CCATGTCTGAGATGATTTCTGAGAAATTGAAAAGTATTTGATGTTGAAGAAGATCAAATGCCCA
GCATGCCCCAGGTAACTTTCAGCAATTGTGGATGCTTAATTCTGTGTTAACACTGAAATTAGAA
TGAAGGGAAAACAGAGTGTGTTGATTTCATGTTGTCAACATAAGGCTGAATTACTCCTACTGAC
ATTGCTGTTGGTTCCAGTAAATGTTTAGAGTGTTTCCATTTCTTCCTACATCATTTACTAACCT
AGGAAGGTTGACCATACCTCAAAAGCTGTATTCTCATGGTGACTGCACTGAAACTTGAGCACAT
TTTATGGAAAACTATTAGGCTCACTCTTTTCATGATCACTGTTTGCTGTGTGTCCTGAGGGCAC
TAATATGACCCCTATCTGGAAGAGTGTCCTTTCACTCCCTCATCAGTGTGTCACCTGGCCAATT
CACTGAGCTCACTTTCTCTGTGTTGTTCCTCTCTGTGAGTAAAAGTCACTTCTGTGTCTTTGGC
TTTCATCCTTTTCTACCTGGCCCTGGTCTATCCCAACATAAAGGCAATAATTTGAGTCCTCATT
AATGGATCTCAGGCTTTTTCTTTTTTAACCACTTCCTTATGTGGAAACCCAGGAAATCTAGCTG
GGGCTCTGTGGTTTCTGATTTTCCCTGGCTCAGGGGCTTCTTTAGTTTTTAAAAAAGAAAAAG
GACTCCATTAATCCAGGCTCAATGGGGTGCTGTTGGGAGTAGAAGGGCCTGAAGAGAAAGACAC
ACACACACACTGAGACTTGCAGGACTCACTGGATAGATGTTATTCGACTCCTGGCCAGGTTACT
GATGAACTACCTGACTCATGCCAGCCCTACAGAAGTGCCTTTTACTCATTGGAGGAACAGCACA
GTGGCTTGGCTCTTGATGAAAACAGTGAGTACAATACTTTTGCATGAAGGTGATAAGGCTCCAC
CTGGTCTTCCAGATAGGGGTGATATTCCAGTTCTAAGTGGCCCTTACTGACCTGAGAGATGTCA
TTGCCAAGGGCAGGACCTATGGGCCTATGTAGGTTGTAATGAAACTGTAGTTTCAGACTTGGAA
GCCCAGACATGAAATGGGTCAGTGAGCATGGCTCTATTCCTAGACTCCAGCCATGCCCAGGTGG
CAACCTGTGCCCACTCTGAATTAATTGGACCCAGGCAGATGTGAAAAATTCACAGGACTATGAT
TTGGACTCAAGGAGTTTGATTTCCTCCTTCATTCTAATTTCAGTGTCTCTGCAAATAAATTCAG
ACAGGGCTGACATACTGCAGTTTTCCTCCTAGCACATCAGGGGCATTTTCTGTGAAATCCTAAC
ACAGGGACATAACTGTTTCCTCAGTGGGAGAAAGGATTTTAGGATAATCTCTACCAGAATAGGG
GATTTCACCCTTGGTTCTGAGATGCCTGAAGAATCTCTGACTGCTTTC

Orangutan m2
AACCAAAGAATCTCTATCATGACCGGCTTTCAGGCCTCCTGAAGTATATCTCTCACATTGTCCT
GTTCTCATGCTGAGGAGCCTGAGATCCCTGTGTGGGGATTAGACAGTGGACTGTTATGGGTGTA
GGTGAATTGGCTTATTTGTCTGTCCCTGTCTGAATTTATTGCAGGAATTAAAAAGGACCAAGA
AGAGGAAGAAGACCAAGGCCCACCATGCCCCAGGTAACTGAGCAATTGTGAACAGCTACTTCTG
TGTTGACACCTGGAGACTCCTGGTTCAGGGAAAACAGAGCAGGCTGACATTATCGATTACATCT
TTCAACCAAGCCTGAATTATTCCTACTAACATTGCTGTTGGTTTTCATTGCAGTAGATATTTAG
GTTTCCATTTCTTCCTCCCCTTATCATTTACTAACCTACTGTAGGTGGACCATACTTCAAAAGC
TGTATTCTCATGGTGACTGCATGGAAACTTGAGCACATTTTATGGAAAATTATTGAGCACAGTC
TTTTCATGATCACTGTACATGTGTCCTGAGGGCACTAACTCAGAGTGTCCTGTTACTCCCTCAT
CAGTGTGTCACCTGGACAATTCACTGAGCTCGTTCTCTCTCTCTCTCTCTGTCTCTGTCTGTGT
GTCTCTCTGTCTGTCTTTCTCTTTCATTCTTTTCCATTTGGCCCTGTTCTGTCCCAAGATGAAG
CCAATAATTTGTTACCTCATTAATGGATCTATCCCTTTTCTTTTTTAACCACTTCCTTATGCTA
CCCATGAAATCTAGTTGGAGCTCTGTTGTGTCTGATTTCCCCTGGCTTATTCTTTACTTTTTCT
ACTTTTCCAGGCTCAGCAGAGAGCTGCTGGAGGTAGTAGAGCCTGAAGAATTGCAGGACTCACT
GGATAGATGTTATTCAACTCCTTCCAGTTATCTTGAACTGCCTGATTTCATGCCAGCCCTATGG
AAGTTCCTTTTACTCATTGGAGGAACAACATGTTGGCTTTTCTCTTGACGTGGATGGTACAGTA
CCTTTCTATGAAGGTGATAAGGATCCACTGAGTCTTCCATATAGAGATCATATTCCTGCTCCAA
GAGGCCATTACTGAGCTGAGAGATGTCATTGCTGCAGTGAGGACCTATAGGCACATGTAGGTTG

AATGAAACTCTAGTTCCAGTTGGAAGCCCAGACATGGGATGGGTGAGTGAGCATGGCTCTCTTC
CTAGTCTCAGGCCATGCCTGTGGCACTCTGATTCTACTCTGAAGACATTGGACCTGTTAGATGT
GACAAATTCACCTAAAGAACTATGATTTTGACTCAAGCAATGTTTGTAGATTTCCTTTTTCACT
CTGAAAAATTTCAGTGTCTAAAATCCTCACAACCATGAACAATCTGAGTATTTGATGAGACAGG
GCTGAATATTGCAGTTTTTCTCCTAGAAATCATTTGAGGGCATTTGCTTTAAATTGATTGGAAA
AATATGGCATAACTTCCTGCACAAACTTGGGACAAATGGGATTGGGATAAGCCAATCTACCAGA
ATAGGGACATTTTACCCACTGTTTCTGGGACAAAAACCAAGGAATCTCTATCATGAGCAGCCTT
CAGG

Orangutan m3
TGCCCAAGGCTCATAAAAGGACTCAAGCCAGTTCTCTCAAGACTTGACCTCAGGCCTACTGGAA
TATTTCTCTCAAAGTCTCCTGTTCTCACACTGACAAGACTGATGTCCCTGTGTTAGGATTAGAC
AGAGGAATGTTTCTGTGTGCAAGGAAGAACTGCTTAATGTAAGAGGCCCCATCTGAATTTATTT
GCAGGACATCGGTGGGATCAAGTGGAAAAGGAGGACCAAGAGGCAACAGGTCCCAGGTGAGTCT
GAGAAATTGTGGACAGTTAATTTGATGTTGACACCTGGAGACGCCAAGTCCAGGGAAAAGAATG
CATGCTGAAAATAATGATTTCATCTTGTCAGACAAGTCTGAATTATGCCTACTAACGTTGCTTT
TGGTTCTCATTACAGTAAATGTTTAAGTTTCCATTTCTTCCTACCCTTATCATTTACTAACCTA
GTGAAGGTTGACCATACCTCCAAAGCTGTATTCTCATGGTGACTGCAGGGAAACCTGAGCACAT
TTTATGCAAAATTATTGAGGCCACACTTTTCATGATCACTGTTCACTGTGTGTCCTGAGGGCAC
AAATAAAGAGTGTCCTTTGACTCCCTCATCAGTGTGTCACCTGGCCAATTCACTGAGCTCACTC
TCTCTCTCTCTCTGTTTGTGTGTGTGTGTGTGTGTATGTGTGTGTGTCTTTCTGTTTCATCCTT
TTCTACCTGGCCCTGGTCTATCCCAACATAAAGGCAATAGTTTGTTACCTCATTAATGGATCTG
TCCTTTTTCTTTTCAAACACTTCCTTATGTTACCCATGAAATCTAGCTGGGGCTGTATGGTTTC
TGTTTCCCCCTGGCTTATTCTTTACTTTTTCCCACTTTTCCAGGCTCAGCAGGGAGCTGCTGGA
TGAGAAAGAGCCTGAAGTCTTGCAGGACTCACTGGATAGATGTTATTCGACTCCTTCAGGTTAT
CTTGAACTGATTGACTTGTGCCAGCCCTACAGAAGTGCCTTTTACGTATTGGAGCAACAGCATG
TTTGCTTGGCTCTTGACGTGGATGGTGAGTAACTTTCTATGAAGGTGATAAGGATCCACTGAGT
CTTCTGGATAGGGTCATATTCCTACTGCAAGTGGCCCTTACTGAGCTGAGAGATGTCGTTGCCA
CAGGGAGGACCTATAGGCACATGTAGGTTGAATGAAACTCTAGTTCCACTTGGAAGCCCAGACA
AGGGATGGGTCAGTGAGCAAGGCTCTCTTCATAGTCTCAGGCCATGTCTGTGGCACCCTAATCC
TACTCTCAAGATGTTGGATCCGGGCAGATGTGACAGATTCACACAACTCTGATTTTGTCTCAAT
TTTGTAGATTTTCTAGATTTCATCCTTCACTCTAATTTCAGCATCTAAAATCCTTGCTACCATG
AACAATCTGAGTATTTGATGAGACAGGGCTGAATATTGCAGTTTTTCTCCTAGAAACCATTTGG
GGGCATTTGCTTTAAATTGATTGGAAAAATATGGCATAACCATTTGCACAAACTTGGGACAAAT
GATATTAGGATAACGATCTACCAGAATAGGGAATTTTATCCACAGTTTCTGGGACAAAAACCAA
GGAATCTCTATCATGATCAGCCTTCGG
